# Retraction Note: High failure rate after Beta-tricalcium phosphate grafting for the treatment of femoral head osteonecrosis: a retrospective analysis

**DOI:** 10.1186/s12891-021-04344-z

**Published:** 2021-05-24

**Authors:** Pei Liu, Xiao-hong Mu, Hua-chen Yu, Jian-lei Guan, Zhao-hui Liu, Wei-guo Wang, Qi-dong Zhang, Wan-shou Guo

**Affiliations:** 1 Beijing University of Chinese Medicine, Yinghuadong Road, Chaoyang District, Beijing, China; 2 Department Orthopedics 4, Beijing University of Chinese Medicine, Dongzhimen Hospital, Beijing, China; 3 Graduate School of Peking Union Medical College, Beijing, China; 4 Department of Orthopaedic Surgery, Beijing Key Lab Immune-Mediated Inflammatory Diseases, China-Japan Friendship Hospital, No. 2, Yinghuadong Road, Chaoyang District, Beijing, 100029 China

**Retraction Note: BMC Musculoskelet Disord 21, 271 (2020)**

**https://doi.org/10.1186/s12891-020-03291-5**

This article has been retracted by the Editor at the request of one of the corresponding authors Wan-shou Guo. Contrary to the statement in the article, the authors did not obtain appropriate ethical oversight for this study. Pei Liu, Hua-chen Yu, Jian-lei Guan, Wei-guo Wang and Qi-dong Zhang agree with this retraction. Xiao-hong Mu and Zhao-hui Liu have not responded to correspondence from the Editor about this retraction.

